# Predictors of pretraumatic stress during the COVID-19 pandemic in Poland

**DOI:** 10.1371/journal.pone.0290151

**Published:** 2023-08-18

**Authors:** Agnieszka E. Łyś, Mirosława Huflejt-Łukasik, Małgorzata Gambin, Anna Studzińska, Kamilla Bargiel-Matusiewicz, Tomasz Oleksy, Anna Wnuk, Daniel Pankowski

**Affiliations:** 1 Faculty of Psychology and Cognitive Science, Adam Mickiewicz University in Poznań, Poznań, Poland; 2 Faculty of Psychology, University of Warsaw, Warsaw, Poland; 3 Toulouse Campus, Icam School of Engineering, Toulouse, France; 4 Faculty of Psychology, University of Economics and Human Sciences in Warsaw, Warsaw, Poland; Beijing University of Technology, CHINA

## Abstract

**Background:**

Pretraumatic stress has the same symptoms as post-traumatic stress but instead pertains to anticipated threats. There is evidence that pretraumatic stress occurs among soldiers and pregnant people.

**Objective:**

We analyzed correlates of pretraumatic stress concerning the threat of COVID-19 infection.

**Method:**

Our pilot study was cross-sectional (*N* = 74); our main study was longitudinal and consisted of three waves (*N* = 1067, *N* = 894, and N = 752 for Waves 1, 2, and 3, respectively). Our pilot study used correlation and multiple linear regression. Our main study used quadratic regression and a random intercept cross-lagged panel model.

**Results:**

The pilot study found that pretraumatic stress was positively correlated with agreeableness (r = .24, *p* < .01) and negatively correlated with emotional stability (r = -.30, *p* < .01) and intellect/imagination (r = -.37, *p* < .01). The main study demonstrated that pretraumatic stress was positively correlated with other measures of mental health problems during the COVID-19 pandemic and with perceived positive aspects of the pandemic (r = .11, *p* < .01). There is evidence of a U-shaped relationship between pretraumatic stress and perceived positive aspects of the pandemic. A random intercept cross-lagged panel model analysis demonstrated that pretraumatic stress in Wave 2 was negatively predicted by levels of prosocial behavior in Wave 1 (B = -1.130, p < .01).

**Conclusion:**

Mental health professionals should take into account pretraumatic stress, not only as a possible consequence of the COVID-19 pandemic outbreak but more generally as a risk in situations that are new, difficult, and challenging for people.

## 1. Introduction

According to the WHO [1], as of 19th July 2023, there have been 768,237, 788 confirmed cases of COVID-19, including 6,951,677 deaths worldwide. Previous studies have suggested that the COVID-19 pandemic profoundly impacts people’s mental health [e.g., 2–7]. The fear and anxiety generated by the possibility of virus spread and contraction, as well as sudden changes and unexpected situations associated with public health instructions and measures for confinement and social and physical distancing associated with the pandemic, could be perceived as serious threats. The threats and stress associated with the COVID-19 crisis may exacerbate negative emotions like sadness, anxiety, anger, and hostility that, in turn, may lead to the perception of the COVID-19 crisis as increasingly threatening, creating a cycle of threat and distress, anxiety, anger, and hostility influence perceived threat of COVID-19 [8].

There is a link between the COVID-19 pandemic and an increase in distressing feelings, including perceived threat of COVID-19 [2–11]. During the COVID-19 pandemic, fear manifests in feelings of loneliness, uncertainty, anxiety, and even panic [12, 13]. Long-term confrontation with the threatening situation generated by the epidemic not only aggravates depression symptoms (in some age groups more than others) but is also related to self-destructive behaviors, such as increased alcohol consumption [14].

Previous studies [e.g., 10, 11] have suggested that the COVID-19 pandemic affects people’s mental health, not just those who have contracted COVID-19. That suggests that even the threat of COVID-19 infection may be traumatic. The results demonstrated by Bridgland et al. [15] seem to support this assumption: their study demonstrated that PTSD-like symptoms during the COVID-19 pandemic were associated not only with experienced traumatic events but also with anticipated ones.

### 1.1. Pretraumatic stress

Pretraumatic stress is defined as *disturbing future-oriented cognitions and imaginations as measured in terms of a direct temporal reversal of the conceptualizations of past-directed cognitions in the PTSD diagnosis* [16, p. 1]. According to a study conducted with 610 Danish soldiers deployed to Afghanistan, pretraumatic stress before deployment was positively correlated with neuroticism and depression symptoms and was a significant predictor of posttraumatic stress after deployment [16].

Goutaudier et al. [17] showed that pretraumatic stress is also a problem for pregnant people awaiting childbirth–in one study with 102 pregnant people, 8.8% had symptoms of traumatic stress related to the delivery. Pretraumatic stress was positively correlated with depressive symptoms, anxiety, anticipated labor pain, fear of delivery [17], eating disorders [18], and worry about one’s self [19].

Furthermore, some researchers [20, 21] suggest that anxiety about climate change is a form of pretraumatic stress, as climate change may cause symptoms of PTSD, compounded stress, substance abuse, and depression [22].

Dragan et al. [10] found that, at the beginning of the pandemic, 75.8% of Polish people examined (*N* = 1742) perceived it as a stressful situation. More than 90% had at least one posttraumatic stress symptom, and 2.4% met the presumptive diagnostic criteria of PTSD. These statistics suggest that pretraumatic stress may also occur in the context of the pandemic. Prete et al. [23] demonstrated that the level of pretraumatic stress during the COVID-19 pandemic in Italy was a significant predictor of affective worry related to the pandemic, which suggests that the level of pretraumatic stress may be an adequate indicator of distress during societal crises such as a pandemic.Specialists (e.g. [24, 25]) identify pretraumatic stress as one of the challenges that medical professionals and medical students can face during the pandemic.

In the current studies, we focus on identifying various predictors of pretraumatic stress during the COVID-19 pandemic in Poland. Firstly, given that neuroticism is positively correlated with pretraumatic stress symptoms [15] as well as with other symptoms of common mental disorders [16], we hypothesized that pretraumatic stress during the pandemic would also be positively correlated with neuroticism (here operationalized as a low level of emotional stability). Additionally, we focused on analyzing the relations of pretraumatic stress to prosocial behavior and perceived positive aspects in the context of changes caused by the pandemic.

#### 1.1.1. Pretraumatic stress and prosocial behavior

There is some evidence that people become more prosocial during societal crises, like wars [26] and natural disasters [27]. Hellmann et al. [28] demonstrated that the endorsement of prosocial values among German people increased at the beginning of the COVID-19 pandemic. There is also some evidence [29–34] of a link between prosocial behavior and psychological well-being during the COVID-19 pandemic. The literature indicates that positive emotions increase one’s proclivity towards prosocial behavior [35, 36; for a review, see: 37]. Some studies [38–44] suggest a reversed cause-and-effect relationship in which prosocial behavior promotes well-being and happiness. We might therefore hypothesize that people who engaged in prosocial behaviors during the pandemic will have had lower levels of distress. On the other hand, prosocial behavior during the pandemic may be linked with the perceived threat, which may account for the need to protect other people against the consequences of the pandemic. To the best of our knowledge, there are no studies on the relationship between prosocial behavior and pretraumatic stress. Our current studies aim to examine this possible relationship. Considering the link between agreeableness and prosocial behavior [45–47], we also decided to investigate whether agreeableness predicts pretraumatic stress and whether prosocial behavior mediates this relationship.

### 1.2. Perceived positive aspects in the context of changes caused by the pandemic

The perception of positive aspects in a stressful situation plays a vital role in self-regulation. Optimism and directing attention towards positive aspects bolsters one’s activity and aspirations and increases motivation when the situation worsens, and stress factors increase [48–52]. Some kinds of interventions can direct one’s attention towards positive aspects, giving meaning to difficult situations [e.g., 53, 54]. Data has shown the importance of identifying positive aspects during significant life changes. Changes can have negative consequences, such as increased psychopathological symptoms (including depressive symptoms, disrupted self-image, and paranoid thinking), and somatic diseases [e.g., colds, the flu; 55–58]. However, people who perceive changes as "for the better" report improved functioning, while those who see changes as "for the worse" report worse functioning. Noticing positive aspects, the ability to pursue important personal goals, social support, and sharing experiences with others while undergoing change can act as buffers against potential adverse effects [59, 60]. Significant increases in subjective life satisfaction during COVID-19 were related to active coping and positive reframing as well as to a person’s assessment of the extent to which something can be done about negative situations, while positive affect was positively related to perceiving the situation as a challenge [61]. Creating a positive possible self is associated with overcoming crises [62], and, after positive life changes have occurred, the proportion of positive content in the future ideal-self increases [63]. Positive-self guides allow individuals to adapt to situations and stabilize their image of themselves [e.g., 64, 65]. In summary, significant changes have a psychological cost and are a challenge. However, the abilities to identify positive aspects, to create positive beliefs about current experiences, and to imagine possible future positive effects help counteract the negative impact of events and life changes. They may thereby decrease levels of pretraumatic stress.

### 1.3. Current studies

These studies sought to identify the predictors of pretraumatic stress and changes in pretraumatic stress over time. Informed consent was obtained in both studies from all participants: after reading the information concerning the study, participants were asked whether they agreed to participate. Only persons who chose the answer “yes” were included in the study. The previously unpublished tools used in this study are available here: https://osf.io/z9vup/

## 2. Pilot study

Drawing on the aforementioned research, we tested the following hypotheses:

H1: Pretraumatic stress symptoms will be negatively predicted by emotional stability.
H2: Pretraumatic stress symptoms will be negatively predicted by prosocial behavior.

We also included other Big Five factors; however, we performed an exploratory analysis as there are no consistent findings concerning their links with pretraumatic stress.

### 2.1. Participants and procedure

We used data from a study on conspiracy theories, registered here: https://osf.io/z9vup/. The study was conducted in April 2020, at the beginning of the COVID-19 pandemic in Poland. The only inclusion criterion was being at least 18 years old, constituting legal adulthood in Poland. The sample consisted of 97 participants; however, 23 were non-native Polish speakers. Thus our final sample consisted of 74 students, including 44 men (60%), 29 women (39%), and one person who described themselves as non-binary. The participants were students recruited from the participants of a social communication course at the University of Economics and Human Sciences. Participants who finished the study obtained extra credits for the course. The mean age of the participants was 25.09 (*SD* = 6.06). The sample comprised 25 psychology students (34%) and 49 computer science students (66%).

Participants completed the questionnaires on Qualtrics. Participation was voluntary, but students obtained course credits. Only persons who gave informed consent participated in the study. At the beginning of the study, the participants read information concerning the purpose of the study, the approximate time it would take to complete the survey, and a brief outline of the study; they were then asked to consent to participate. Only persons who gave informed consent participated in the study. The participants filled in the questionnaires twice, but the hypotheses in this paper concern only the first measurement. Our project was approved by the Ethical Review Board at the Faculty of Psychology at the University of Warsaw.

### 2.2. Measures

#### 2.2.1. Pretraumatic stress related to the COVID-19 pandemic

We translated the Pretraumatic Stress Reactions Checklist [16] into Polish. The Polish version was then back-translated, and some minor changes were made. The items were modified so that they pertained to possible COVID-19 infection (e.g., Feeling very upset when something reminded you of a possible future COVID-19 infection instead of Feeling very upset when something reminded you of a possible future stressful experience). The scale consisted of 20 items, 9 of which concerned trauma symptoms related to possible future COVID-19 infection (sample item: *Repeated*, *disturbing dreams of a possible future* COVID-19 *infection*) and 11 of which concerned trauma symptoms where the time and character of the traumatic event was not precise (e.g., *Loss of interest in activities that you used to enjoy*). The internal consistency in the sample was α = .93.

#### 2.2.2. Prosocial behaviors during the COVID-19 pandemic

W**e** constructed a questionnaire that assessed how frequently, during the pandemic, participants engaged in 12 prosocial behaviors from four categories: emotional support, informational support, appraisal, and instrumental support [66]. Emotional support includes verbal and nonverbal messages of positive feelings toward someone (e.g., *A conversation with somebody who needs to share their difficult emotions elicited by the pandemic or its consequences*); informational support includes giving advice and information intended to facilitate problem-solving (e.g., *Giving another person information on how to prevent COVID-19 infection*); appraisal involves the communication of acknowledgment and appreciation (e.g., *Expressing support for somebody who spread reliable information about COVID-19*); while instrumental support constitutes practical help like financial support or helping with day-to-day tasks (e.g., *Helping a person who cannot leave their house because of the pandemic with their daily chores*). The internal consistency in the sample was α = .82 for the whole scale. Due to the low internal consistency of the instrumental support subscale (α = .50), we analyzed the total of the scores without division into subscales.

#### 2.2.3. Big five personality traits

We measured the Big Five personality traits with the 20-item Big Five Markers questionnaire from the International Personality Item Pool [67]. The Polish version was prepared by Topolewska et al. [68]. It has five subscales, consistent with the Big Five factors: emotional stability, which is the opposite of neuroticism (e.g., *I have frequent mood swings;* α = .62); extraversion (e.g., *I talk to a lot of different people at parties; α =* .86); agreeableness (e.g., *I sympathize with others’ feelings;* α = .70); conscientiousness (e.g., *I like order;* α = .70); and intellect/imagination (e.g., *I have a vivid imagination;* α = .59).

#### 2.2.4. Social desirability bias

We used a brief version of the Marlowe–Crowne Social Desirability Scale [69, 70], prepared by Janowski and Piasecka [71], to measure social desirability. It consists of 7 items (e.g., *I never resent being asked to return a favor*) on a Likert scale from 1 (*definitely no*) to 4 (*definitely yes*). Its internal consistency in the sample was α = .68.

#### 2.2.5. Sociodemographic data

We asked participants about their gender, age, field of study, and professional situation. We also asked them about details related to the pandemic: whether they had been infected by COVID-19, whether they knew somebody who had been infected by COVID-19, and whether they had been quarantined.

### 2.3. Statistical analysis

In order to test H1 and H2, we used r-Pearson correlation and multiple linear regression. The analyses were performed with SPSS 21.

### 2.4. Results of the pilot study

#### 2.4.1. Descriptive statistics

Descriptive statistics are presented in S1 Table, which is in the Supplementary Materials.

None of the variables reached an absolute value of skewness higher than two nor an absolute value of kurtosis higher than 7. Thus the assumption of normality of the distribution was not violated [72, 73].

#### 2.4.2. Correlation matrix

Partial correlations (controlling for social desirability) between pretraumatic stress and other variables are presented in S2 Table, which is in Supplementary Materials.

Pretraumatic stress was positively correlated with agreeableness and negatively correlated with emotional stability and intellect/imagination. Unexpectedly, pretraumatic stress turned out to be positively correlated with prosocial behavior.

#### 2.4.3. Predictors of pretraumatic stress–multiple regression

We conducted multiple regression in order to identify predictors of pretraumatic stress. To remove outliers, we excluded 23 observations with either Cook’s distance above 1, Leverage above (k+1)/n, or Mahalanobis distance above 15 [74, 75]. In order to control multicollinearity, we performed a series of Variable Inflation Factors (VIF) tests. Their results ranged from 1.146 for intellect to 1.321 for agreeableness, which is an acceptable value [76]. We also conducted a post-hoc power analysis with the Post-hoc Statistical Power Calculator for Multiple Regression [77]. It turned out that the power was 1-β = .93, which is a good score. The results of the multiple regression analysis are presented in Table 1.

**Table 1:** Predictors of pretraumatic stress: Multiple regression (pilot study).

	β	*t*
Intercept		2.752
Emotional stability	.22	1.82
agreeableness	-20	-1.64
Intellect/imagination	-.37**	-3.23
Prosocial behavior	.22*	1.92
Social desirability	.01	.02

*p < = .05

**p < = .01d

Emotional stability and agreeableness were no longer significant predictors of pretraumatic stress, whereas intellect/imagination and prosocial behavior remained significant. The R2 of the model was .29, which means that 29% of the variance in pretraumatic stress can be explained with this model.

### 2.5. Discussion of the pilot study

Hypothesis 1 was supported, as emotional stability was positively associated with pretraumatic stress. Hypothesis 2 was not supported because prosocial behavior positively predicted pretraumatic stress. The positive correlation between prosocial behavior and pretraumatic stress is puzzling, especially since, according to our regression model, prosocial behavior positively predicted pretraumatic stress, even after taking into account emotional stability, agreeableness, and social desirability. Prosocial behavior may be used as a tool for reducing negative emotions. Studies demonstrating that prosocial behaviors promote happiness [38–44] support this conclusion. We should also consider that people who are in a positive mood are not always likely to help others. Isen and Simmonds [78] found that people in a positive mood are less inclined to help others when they perceive the task as inducing negative emotions. Therefore, people in a relatively good mood during the pandemic may not have been inclined to help others with their pandemic-related problems for fear of increased distress. In contrast, people experiencing negative emotions may not experience such inhibition.

Nonetheless, we cannot tell whether prosocial behavior influenced the participants’ well-being because our study was correlational. In order to draw any cause-and-effect conclusions, we would have to use a model based on repeated measures, such as a random intercept cross-lagged panel model [79]. Another significant limitation of the study was its small and non-representative sample. We sought to overcome these limitations in our main study.

## 3. Main study

The main study aimed to investigate associations between pretraumatic stress, other measures of distress and various risk and protective factors. We took social support into account, as close relationships with others are essential for buffering the adverse effects of changes [60].

There is some evidence that social support is vital for alleviating symptoms of depression [80] and promoting posttraumatic growth [81–84]. However, to our knowledge, no existing studies investigated a link between social support and pretraumatic stress. Therefore, we decided to examine whether social support helps to reduce pretraumatic stress. We also investigated whether levels of pretraumatic stress decrease with time. We predicted that people might become more familiar with the COVID-19 threat as time passes, and thus desensitization might occur.

We also investigated whether the unexpected positive correlation between pretraumatic stress and prosocial behavior found in the pilot study can be replicated in a bigger and more representative sample. Based on existing evidence that, in certain situations, providing support has a greater effect on well-being than receiving support [85, 86], we assumed that this effect would be observed in a study conducted on a bigger sample. Specifically, we expected that prosocial behavior in Wave 1 would negatively predict pretraumatic stress in Wave 2 and prosocial behavior in Wave 2 would negatively predict pretraumatic stress in Wave 3. Furthermore, we examined whether prosocial behavior and pretraumatic stress correlated with the perceived risk of COVID-19. People with a high perceived risk of COVID-19 may experience greater stress levels, and thus they may have more pretraumatic stress symptoms. They may also be more inclined towards prosocial behaviors because they may want to protect other people from the effects of the pandemic.

Finally, we also investigated whether pretraumatic stress correlated with the pandemic’s perceived positive aspects. According to Tedeschi and Calhoun [87], post-traumatic growth is not simply a measure of mental health. They suggest that the link between post-traumatic growth and psychological fitness may be curvilinear. People who experience low levels of distress may not significantly change their functioning due to a traumatic event, and they may, therefore, not experience the positive effects of traumatic events. On the other hand, people who experience a high level of distress may be unable to develop strategies that stimulate post-traumatic growth. Studying post-traumatic growth related to the pandemic was not possible in 2020, as the pandemic was an ongoing event. Therefore, we decided to study the perceived positive aspects of the pandemic. We hypothesized that the relationship between pretraumatic stress and perceived positive aspects of the pandemic would be curvilinear, similar to the relationship between distress and post-traumatic growth in previous studies [87].

We tested the following hypotheses:

H1: The relationship between pretraumatic stress and perceived positive aspects of the pandemic will have an inverted U- shape—people with moderate levels of pretraumatic stress will see more positive aspects of the pandemic than people with low and high levels of pretraumatic stress.
H2: Pretraumatic stress in Wave 2 will be lower than in Wave 1 (H7a), and pretraumatic stress in Wave 3 will be lower than in Wave 2 (H7b).
H3: Prosocial behavior in Wave 1 will negatively predict pretraumatic stress in Wave 2 (H8a), and prosocial behavior in Wave 2 will negatively predict pretraumatic stress in Wave 3 (H8b).
H4: Received social support in Wave 1 will negatively predict pretraumatic stress in Wave 2 (H9a), and received social support in Wave 2 will negatively predict pretraumatic stress in Wave 3 (H9b).

### 3.1. Participants and procedure

The study was conducted in three waves between the 4th of May and the 17th of July 2020. The sample from Wave 1 was representative of the Polish population in terms of gender, age, and place of residence. The participants were recruited from the Ariadna Internet panel, on which people are rewarded for taking part in studies with points that can be exchanged for gifts. The only inclusion criteria were being at least 18 years old, which is legal adulthood in Poland, and not having been infected by COVID-19. A total of 1,100 persons participated in Wave 1, but 33 of them had already been infected by COVID-19; thus, the final sample consisted of 1067 participants. The mean ages of the participants were 45.02 (*SD* = 15.76), 46.72 (*SD* = 15.18), and 47.16 (*SD* = 14.87) in Waves 1, 2, and 3, respectively. Further demographic characteristics of the sample are shown in S3 Table, which is in the Supplementary Materials.

The first wave occurred between the 4th and 7th of May–two months after detecting the first case of COVID-19 in Poland. Strict restrictions had been implemented six weeks before the first wave of our study, including the suspension of universities, schools, preschools, nurseries and a ban on movement except for essential activities. The most stringent restrictions started to be gradually lifted from the 20th of April.

The second wave took place between the 4th and 17th of June. The gradual lifting of restrictions was continued, and almost all restrictions introduced to contain the pandemic were removed. The Polish prime minister emphasized that Poland was emerging victorious in the fight against the epidemic [88]. Data for the third wave were gathered between the 7th and 17th of July. Outbreaks occurred in hospitals, workplaces, weddings, baptisms, funerals, and even holiday centers. The second round of the presidential elections took placein Poland. The Prime Minister assured the public that the COVID-19 situation was stabilizing. Our project was approved by the Ethical Review Board at the Faculty of Psychology at the University of Warsaw.

### 3.2. Measures

#### 3.2.1. Pretraumatic stress related to the COVID-19 pandemic

We used the same tool as in the pilot study. The internal consistencies were α = .97, α = .97, and α = .98 for Waves 1, 2, and 3, respectively.

#### 3.2.2. Prosocial behavior during the COVID-19 pandemic

We chose the eight items from the scale used in the pilot study with the highest item-total correlation and one new item. The internal consistencies in the sample were α = .87, α = .90, and α = .91 in Waves 1, 2, and 3, respectively.

#### 3.2.3. Perceived risk of COVID-19

We used a scale consisting of six questions about the subjective risk of (i) COVID-19 infection, (ii) serious adverse health effects and complications due to COVID-19 infection, and (iii) threat to life as a result of infection. Each of the three risk areas was assessed using two items, one relating to oneself and the other to close relatives and loved ones. Items were rated on a five-point scale ranging from 1 (*very low*) to 5 (*very high*). The internal consistencies of the total scale in the sample were α = .92, α = .94, and α = .95 in Waves 1, 2, and 3, respectively.

#### 3.2.4. Received social support

We used a scale consisting of five questions about three kinds of social support, basing our items on the brief version of the Medical Outcomes Study Social Support Survey [89] (i) emotional–informational support, (ii) tangible support and positive social interaction, and (iii) affectionate support. Participants indicated to what extent they had access to each form of support on a scale from 1 (*definitely not*) to 5 (*definitely yes*). The internal consistencies of the total scale in the sample were α = .75, α = .77, and α = .79 in Waves 1, 2, and 3, respectively.

#### 3.2.5. Depression symptoms

We used the Patient Health Questionnaire-9 (PHQ-9: [90]; Polish version: [91])–a screening tool for assessing the risk of depression, consisting of nine questions concerning depression symptoms (e.g., Little interest or pleasure in doing things). Participants indicated how often they had experienced each symptom during the two weeks before the study on a scale from 0 (not at all) to 3 (nearly every day). The internal consistencies of the total scale in the sample were α = .89, α = .92, and α = .92 in Waves 1, 2, and 3, respectively.

#### 3.2.6. Anxiety symptoms

We used the Generalized Anxiety Disorder-7 (GAD-7: [92]; Polish version: [91])–a screening tool for assessing the risk of general anxiety disorder, consisting of seven questions concerning general anxiety disorder symptoms (e.g., Feeling nervous, anxious, or on edge). Participants indicated how often they had experienced each symptom during the two weeks before the study on a scale from 0 (not at all) to 3 (nearly every day). The internal consistencies of the total scale in the sample were α = .94, α = .96, and α = .96 in Waves 1, 2, and 3, respectively.

#### 3.2.7. Perceived positive aspects of the pandemic

We prepared a scale consisting of eight items concerning perceived positive aspects of the pandemic (sample item: *I see also the positive aspects*, *not only the difficulties of the epidemic situation*) on a Likert scale from 1 (*definitely no*) to 5 (*definitely yes*). We used this scale only in Wave 1. The internal consistency in the sample was α = .89.

#### 3.2.8. Sociodemographic data

We asked participants about their gender, age, field of study, and professional situation. We also asked them about details related to the pandemic: whether they had been infected by COVID-19, whether they knew somebody infected by COVID-19, and whether they had been quarantined.

### 3.3. Statistical analysis

First, we checked the correlations between the variables with the r-Pearson correlation coefficient. In order to test H1, we used quadratic regression. In order to test H2, we used repeated-measures ANOVA. In order to test H3 and H4, we used the random intercept cross-lagged panel model (RI-CLPM). The RI-CLPM was performed with the powRICLPM [93] and lavaan packages in R 4.1.0 [94]. Other analyses were performed with SPSS 21.

### 3.4. Results of the main study

#### 3.4.1. Attrition analysis

In order to check whether there are any systematic patterns of attrition, we compared incomplete responders (n = 324) to complete responders (n = 743) in terms of key demographic variables and the main variables used in the models. Among people who completed the study, there were significantly more men than was to be expected, χ2(1) = 8.136, p = .004, and significantly more people with a university degree than was to be expected, χ2(1) = 4.685, p = .030. People who completed the study were significantly older than those who did not, t(560) = 6.888, p < = .001. It turned out that people who completed the study had lower levels of depressive symptoms, t(1065) = -3.660, p < .001, and higher levels of perceived COVID-19 risk, t(1065) = 2.093, p = .037, in Wave 1 than people who did not complete the study. There were no significant differences in pretraumatic stress, t(1065) = 0.170, p = .865, generalized anxiety, t(1065) = -1.770, p = .077, perceived positive aspects of the pandemic, t(1065) = -1.659, p = .097, prosocial behavior, t(1065) = -1.550, p = .122, or received social support, t(1065) = -0.430, p = .667, in Wave 1 between complete and incomplete participants. We then performed a logistic regression to check whether the completion of the study was predicted by any of the aforementioned variables. The model explains only 6% of the variance in attrition (R2 Cox and Snell = .06). The attrition was predicted only by age, Wald χ2(1) = 33.931, p < .001, and gender, Wald χ2(1) = 6.763, p = .009. Thus we decided to control those variables in our analyses in order to be able to treat the missing data as missing at random (see [95]), which means that although there might be systematic differences between the missing and observed values, we can entirely explain them with other observed variables [96]. In order to obtain unbiased parameter estimates despite the missing data, we used full information maximum likelihood [97].

#### 3.4.2. Descriptive statistics

Descriptive statistics are presented in S4 Table, which is in Supplementary Materials.

**N**one of the variables reached an absolute value of skewness higher than two nor an absolute value of kurtosis higher than 7. Thus, the assumption of normality of the distribution was not violated [72, 73].

#### 3.4.3. Correlation matrix

Correlations of pretraumatic stress with other variables are presented in S5 Table, which is in Supplementary Materials.

In all three waves, pretraumatic stress was positively correlated with depressive symptoms, generalized anxiety symptoms, perceived risk of COVID-19, and prosocial behavior. rosocial behavior was positively correlated with perceived risk of COVID-19. Pretraumatic stress was positively correlated with received social support in Wave 1, but the correlation was low.

#### 3.4.4. Pretraumatic stress and perceived positive aspects of the pandemic

**Fig 1:**
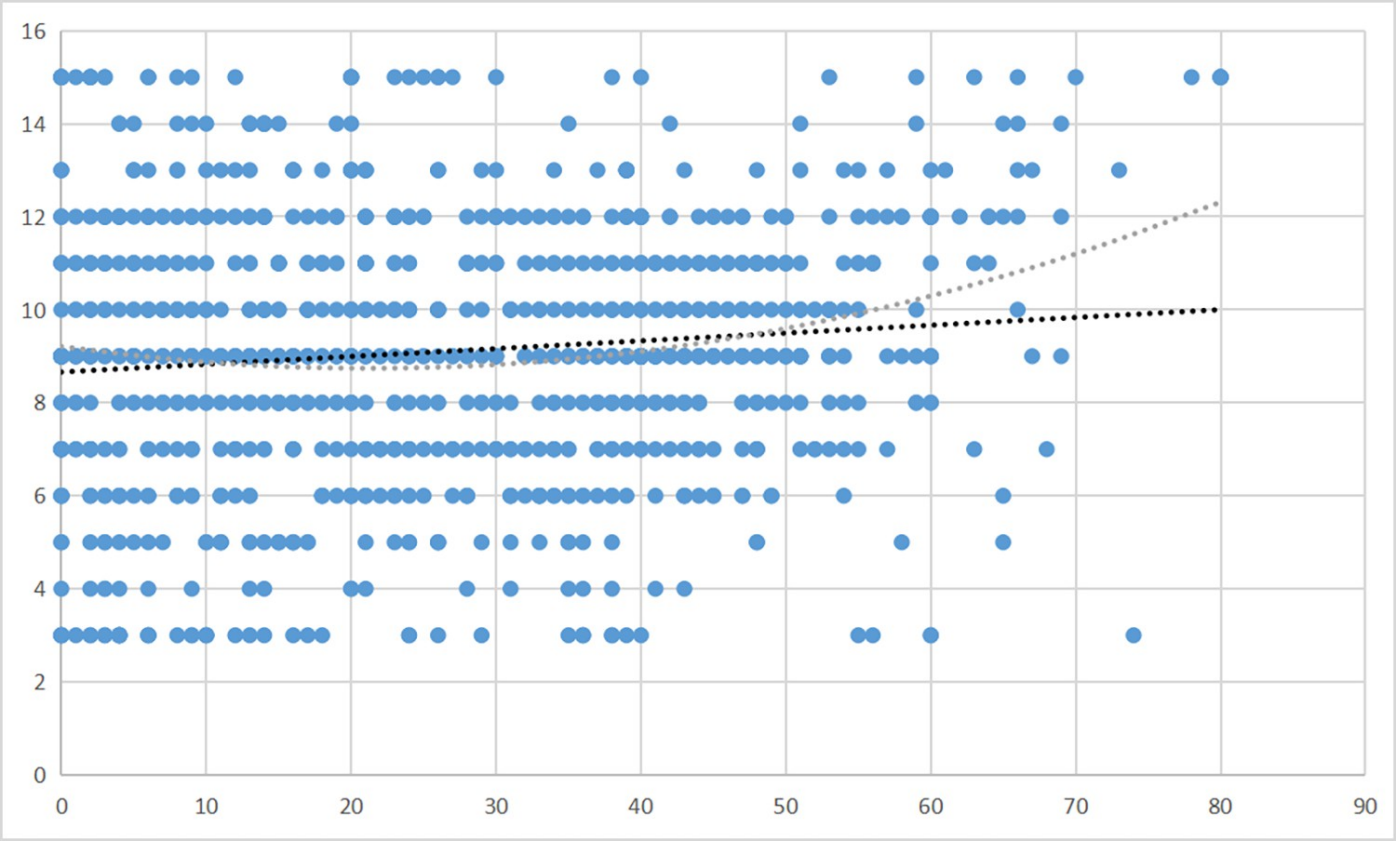
Pretraumatic stress and perceived positive aspects of the pandemic.

We tested a linear relationship between pretraumatic stress and perceived positive aspects of the pandemic in Model 1 and a quadratic relationship between these variables in Model 2. The results are presented in Table 2 and Fig 1.

**Table 2:** Pretraumatic stress as a predictor of perceived positive aspects of the pandemic: Linear and curvilinear regression.

	Model 1			Model 2		
Predictor variable	*B*	β	*t*	*B*	β	*t*
Intercept	22.94		64.40***	23.96		50.72***
Pretraumatic stress	.038	.106	3.47***	-.08	-.212	-2.08*
Pretraumatic stress^2^				.01	.332	3.26**

*p < = .05

**p < = .01

***p < = .001

The curvilinear relationship was significant; however, it was the opposite of what was predicted–it was U-shaped rather than an inverted U-shape. This result means that people with low and high (but not medium) levels of pretraumatic stress saw the most positive aspects of the pandemic. However, the R2 of the model was .02, which means that only 2% of the variance in pretraumatic stress can be explained with this model. We also conducted a post-hoc power analysis with the Post-hoc Statistical Power Calculator for Multiple Regression [77]. The power was 1-β = .99, which is a good score.

#### 3.4.5. Level of pretraumatic stress–longitudinal analysis

In order to compare levels of pretraumatic stress, we conducted a repeated-measures ANOVA. The *F* value was .021, and the significance level was .885. Thus, there were no significant differences in pretraumatic stress levels between the study’s waves. The mean levels of pretraumatic stress in Waves 1, 2, and 3 were 26.87 (*SD* = 17.86), 27.44 (*SD* = 18.10), and 26.79 (*SD* = 18.35), respectively.

#### 3.4.6. Prosocial behavior and pretraumatic stress–longitudinal analysis

**Fig 2:**
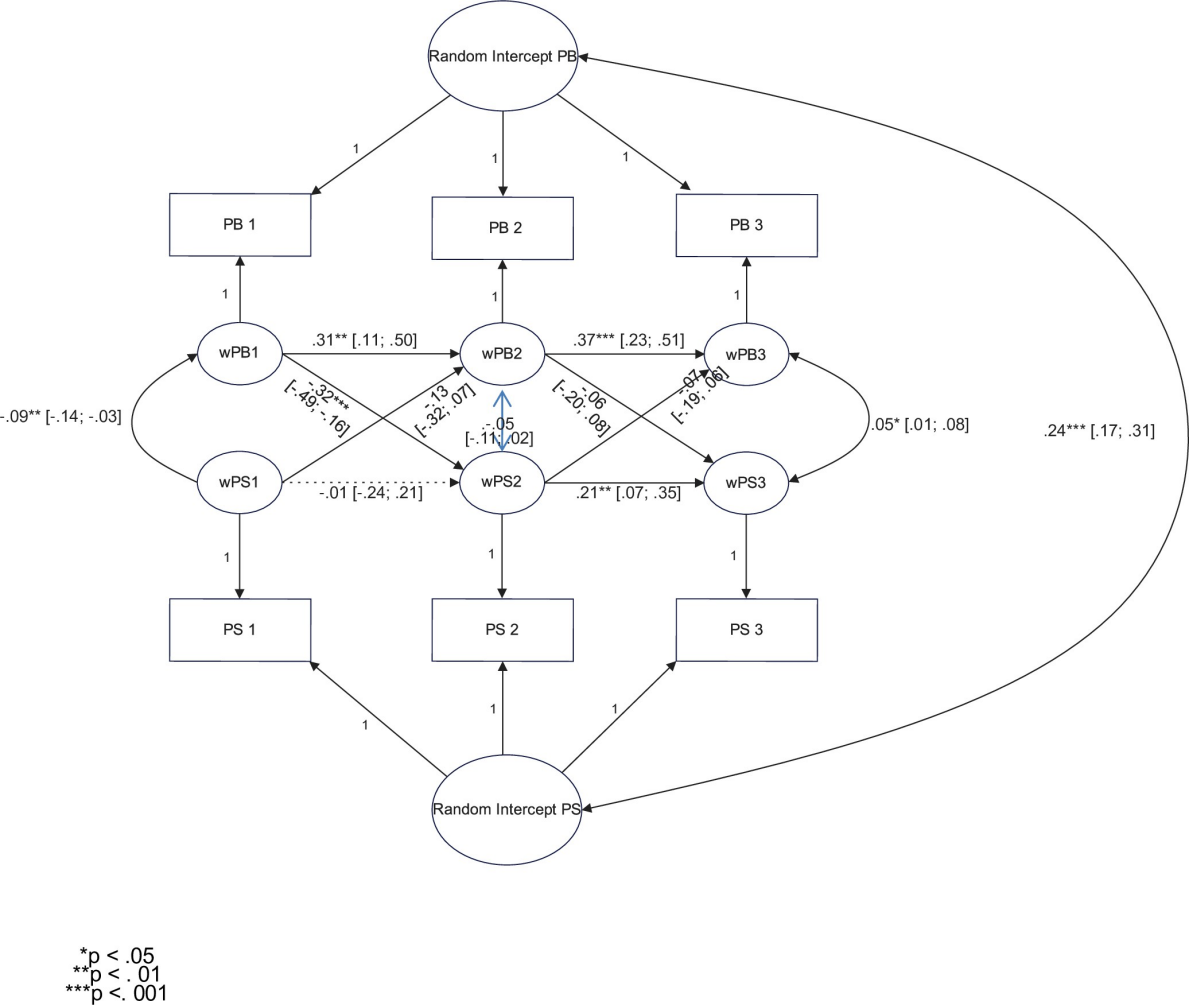
Pretraumatic stress and prosocial behavior: Random intercept cross-lagged panel model. The fit measures were acceptable (based on the parameters from [98]): chi^2^ = 7.411 (df = 1), CFI = .99, TLI = .96, and RMSEA = .08 (CI 95%: .03; .13).

Considering the positive correlation between prosocial behavior and pretraumatic stress in all three waves, we tested the link between prosocial behavior and pretraumatic stress across the waves. We conducted a random intercept cross-lagged panel model analysis to check whether prosocial behavior in Wave 1 predicted pretraumatic stress in Wave 2 and prosocial behavior in Wave 2 predicted pretraumatic stress in Wave 3. We performed the analyses on standardized data. The cross-lagged effects were not constrained. The parameters with 95% confidence intervals are presented in Fig 2.

Pretraumatic stress in Wave 2 was predicted by levels of prosocial behavior in Wave 1. In order to make sure that our analysis had adequate power, we performed a post-hoc power analysis with the *powRICLPM* package in R [93]. We used standardized variables. It turned out that the power for the effect of prosocial behavior in Wave 1 on pretraumatic stress in Wave 2 was 1-β = .98, which is a good score. Nonetheless, pretraumatic stress in Wave 3 was not predicted by levels of prosocial behavior in Wave 2.

#### 3.4.7. Received social support and pretraumatic stress–longitudinal analysis

**Fig 3:**
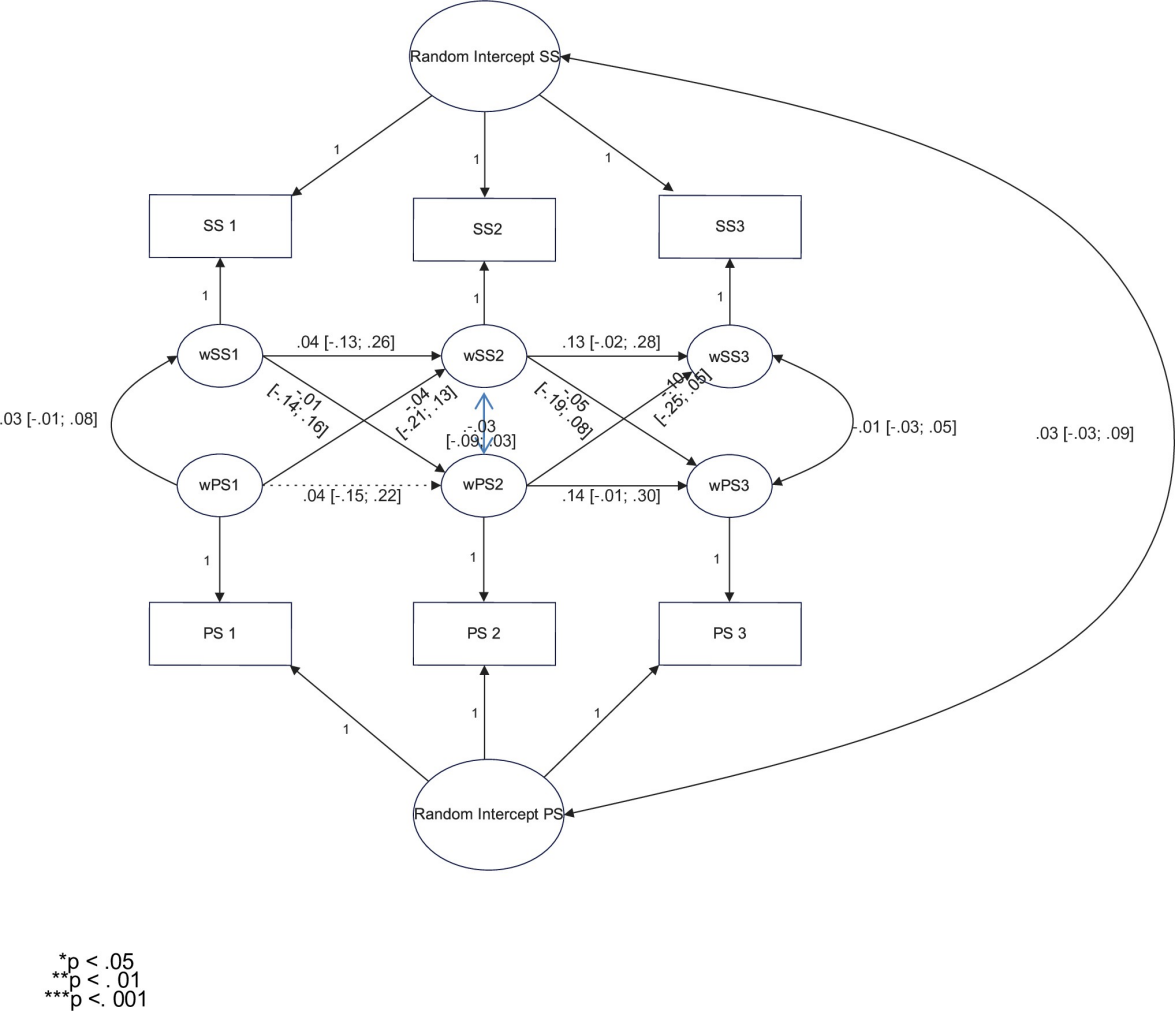
Received social support and pretraumatic stress: Random intercept cross-lagged panel model. The fit measures were very good (based on the parameters from [98]): chi2 = 1.599 (df = 1), CFI = 0.99, TLI = .99, and RMSEA = .02 (95% CI: 0.00–0.08). As can be seen, social support received did not predict pretraumatic stress across waves.

**Considering** the low but positive correlation between received social support and pretraumatic stress in Wave 1, we tested the link between received social support and pretraumatic stress across the waves. We conducted a random intercept cross-lagged panel model analysis to check whether social support received in Wave 1 predicted pretraumatic stress in Wave 2 and social support received in Wave 2 predicted pretraumatic stress in Wave 3. We performed the analyses on standardized data. The cross-lagged effects were not constrained. The parameters with 95% confidence intervals are presented in Fig 3.

### 3.5. Discussion of the main study

Pretraumatic stress was positively correlated with other measures of distress: depressive symptoms, generalized anxiety symptoms, and perceived risk of COVID-19. Counterintuitively, pretraumatic stress was positively correlated with received social support in Wave 1. There was no correlation between these variables in Waves 2 and 3. Pretraumatic stress was negatively correlated with prosocial behavior.

Hypothesis 1 was not confirmed. The result was the opposite of what was expected: people with low and high levels of pretraumatic stress identified the most positive aspects of the pandemic. Hypothesis 2 was not confirmed. There were no differences in levels of pretraumatic stress across the three waves. Hypothesis 3 was partially confirmed. Prosocial behavior in Wave 1 negatively predicted pretraumatic stress in Wave 2; however, there was no such regularity between Wave 2 and Wave 3. Hypothesis 4 was not confirmed. There was no evidence of a link between received social support and pretraumatic stress between the waves.

## 4. General discussion

The negative correlation between pretraumatic stress and emotional stability found in the pilot study as well as the positive correlation between pretraumatic stress and depression, generalized anxiety, and perceived risk of COVID-19 found in the main study, suggest that the Pretraumatic Stress Reactions Checklist is a valid measure of mental health problems during the COVID-19 pandemic. Neuroticism (here operationalized as a low level of emotional stability) was not the only Big Five factor related to pretraumatic stress. Pretraumatic stress was positively correlated with agreeableness and negatively with intellect/imagination. The negative correlation between pretraumatic stress and intellect/imagination can result from a greater ability to cope with stressful situations and use protective mechanisms, like the ability to perceive the positives and sources of development that can come even with complex changes. The positive correlation between agreeableness and pretraumatic stress is also puzzling. It may be explained by the hypothesis that more agreeable people are more concerned about the pandemic affecting their family and friends and consequently may be more prone to pretraumatic stress symptoms. Nonetheless, this hypothesis requires further investigation.

There was also a U-shaped relationship between pretraumatic stress and perceived positive aspects of the pandemic: people with low and high levels of pretraumatic stress see the positive aspects of the pandemic to the greatest extent. Perhaps people with low levels of pretraumatic stress have good mental health, allowing them to see these positive aspects of the pandemic. On the other hand, people with high levels of pretraumatic stress may need to develop cognitive strategies to identify positive aspects of the pandemic. It is easier for a person in a better emotional state to be creative and optimistic, thereby discovering positive outcomes. On the other hand, looking for the positives is, in some contexts, a protective mechanism activated to improve one’s situation, including one’s emotional state. As a result, the person is motivated to take care of themselves in this way or to use a problematic but inevitable situation for their development [99].

It might also be helpful to differentiate between the mechanisms of the two phenomena. Unrealistic optimism–a bias that is quite common and probably universal–operates when threats are distant and uncertain. It decreases when threats become closer and more real [100]. This mechanism could be responsible for perceiving positive aspects when levels of pretraumatic stress are low. When stress levels are high, then perhaps protective self-regulation mechanisms come into play as a reaction to the significant life changes. Actively looking for positive aspects or advantages in such a situation could be one of these self-regulation mechanisms.

There was a positive correlation between pretraumatic stress and prosocial behavior. On the one hand, people who feel that COVID-19 is a threat (and consequently have high levels of pretraumatic stress symptoms) may be more inclined to help others during the pandemic to help them cope with its adverse effects. On the other hand, people who help others cope with the adverse effects of the pandemic are more exposed to negative feelings related to the pandemic. Nonetheless, levels of prosocial behavior in Wave 1 negatively predicted pretraumatic stress in Wave 2. This result is consistent with previous studies that demonstrated the beneficial effects of prosocial behavior on well-being [38–44].

Nonetheless, levels of prosocial behavior in Wave 2 did not predict the level of pretraumatic stress in Wave 3, which suggests that this effect may attenuate over time. This result is consistent with the Social Support Deterioration Deterrence Model of reactions to societal crises [101, 102], where the mobilization phase, where people activate all the accessible resources to help others, is followed by the deterioration phase, where people start to be aware that the societal needs exceed the resources. This awareness may lead to emotional distress. Thus the disappearance of the buffering effect of the prosocial behavior may be related to the deterioration phase.

These results highlight the importance of social relations when pretraumatic stress is on the rise. This constatation is consistent with previous studies that emphasize the role of social connectedness for mental health [103]. Consistent with research from before the pandemic [85, 86], the results also suggest that supporting others may be even more beneficial for mental health than receiving social support.

### 4.1. Limitations and future directions

The sample in the pilot study was small and non-representative. The sample in the main study was much bigger and representative. However, the main study may have been subject to attrition bias. People who were particularly affected by the COVID-19 pandemic might have withdrawn from the study during Waves 2 and 3. The lower level of depressive symptoms in participants who completed all waves than in participants who dropped out seems to support this assumption. We only measured the perceived positive aspects of the pandemic in Wave 1. herefore we cannot conclude the cause-and-effect relationship between this variable and other variables.

Both studies were conducted in one country—Poland. However, the level of prosocial behavior differs across countries and cultures. Luria et al. [104] demonstrated that prosocial behavior is predicted by some of the Hofstede dimensions—positively by individualism and negatively by power distance, uncertainty avoidance, and long-term orientation. Countries and cultures differ in the level of pandemic-related distress as well. Kowal et al. [105] demonstrated that country-level mortality from COVID-19 predicted the level of distress at the beginning of the pandemic. According to data from 10th March 2023 [106], the mortality from COVID-19 in Poland is 314.45 deaths per 100 000 citizens. It would be interesting to compare Poland with countries with high mortality, like, e.g., Peru (665.84 deaths per 100 000 citizens), and low mortality, e.g., New Zealand (52.88 deaths per 100 000 citizens).

The U-shaped relationship between pretraumatic stress and perceived positive aspects of the pandemic warrants further research. There is some evidence that coping strategies, such as acceptance [107], spirituality [108], religious coping [107, 108], benefit finding [109], and positive reappraisal [107–109], stimulate post-traumatic growth. Recent studies [110–119] demonstrate that coping strategies play a crucial role in the emergence of post-traumatic growth during the COVID-19 pandemic. It would be worthwhile to investigate whether they also stimulate the perception of positive aspects of the pandemic. It will also be possible to examine the predictors of pandemic-related post-traumatic growth once the pandemic is over.

The relationship between pretraumatic stress and the Big Five personality traits also needs further investigation. Intellect/imagination may help develop coping strategies. Therefore, examining the relationship between pretraumatic stress, personality, and coping strategies during the pandemic would be worthwhile

### 4.2. Practical implications and conclusion

Despite the pandemic having effectively ended, new cases are still being recorded in many countries, and the virus still threatens public health. For example, during the week between the 17th and 23rd July 2023, as many as 491 people died because of COVID-19 [1]. In addition, there is some evidence that people affected by COVID-19 struggle with mental health difficulties for a long time [120, 121]. A better understanding of the psychological mechanisms determining levels of mental health can bring many benefits, not only from a theoretical perspective but also from a practical point of view. The results of the research presented above can be used in planning interventions–both directly, such as through psychoeducation, and indirectly, such as through publicity campaigns directed at the general public or populations more exposed to negative psychological consequences. In particular, our results suggest that interventions focusing on promoting prosocial behaviors and concern towards others could help increase resilience and prevent pretraumatic stress, during the COVID-19 pandemic and other social crises. Similarly, perceiving positive aspects of the crisis is an important strategy involved in self-regulation processes that helps a person achieve greater well-being in the face of demanding life challenges and changes.

Pretraumatic stress was an important component of mental health problems during the COVID-19 pandemic. Therefore mental health professionals should take it into account when planning and conducting interventions in the context of COVID-19 and other stressful and challenging life events.

## Supporting information

S1 Table Descriptive statistics (pilot study). *p < = .05 **p < = .01 ps–Pretraumatic Stress, pb–Prosocial Behavior, es–Emotional Stability, ext–Extraversion, agr–Agreeableness, con–Conscientiousness, int–Intellect/Imagination, sd–Social Desirability.
(DOCX)

S2 Table Correlates of pretraumatic stress (pilot study), controlling for social desirability. *p < = .05 **p < = .01 ps–Pretraumatic Stress, pb–Prosocial Behavior, es–Emotional Stability, ext–Extraversion, agr–Agreeableness, con–Conscientiousness, int–Intellect/Imagination.
(DOCX)

S3 Table Sociodemographic characteristics (main study). (DOCX)

S4 Table Descriptive statistics (main study). ps1 –Pretraumatic Stress (Wave 1), d1 –Depressive Symptoms (Wave 1), gad1 –Generalized Anxiety Disorder (Wave 1), ss1 –Social Support (Wave 1), pb1 –Prosocial Behavior (Wave 1), pr 1 –Perceived Risk of COVID-19 (Wave 1), pp 1– Perceived Positive Sides of Pandemic (Wave 1), ps2 –Pretraumatic Stress (Wave 2), d2 –Depressive Symptoms (Wave 2), gad2 –Generalized Anxiety Disorder (Wave 2), ss–Social Support (Wave 2), pb–Prosocial Behavior (Wave 2), pr2 –Perceived Risk of COVID-19 (Wave 2), ps3 –Pretraumatic Stress (Wave 3), d3 –Depressive Symptoms (Wave 3), gad3 –Generalized Anxiety Disorder (Wave 3), ss3 –Social Support (Wave 3), pb3 –Prosocial Behavior (Wave 3), pr3 –Perceived Risk of COVID-19 (Wave 3).
(DOCX)

S5 Table Correlates of pretraumatic stress. *p < = .05 **p < = .01, ps1 –Pretraumatic Stress (Wave 1), d1 –Depressive Symptoms (Wave 1), gad1 –Generalized Anxiety Disorder (Wave 1), ss1 –Social Support (Wave 1), pb1 –Prosocial Behavior (Wave 1), pp 1– Perceived Positive Sides of Pandemic (Wave 1), pr 1 –Perceived Risk of COVID-19 (Wave 1), ps2 –Pretraumatic Stress (Wave 2), d2 –Depressive Symptoms (Wave 2), gad2 –Generalized Anxiety Disorder (Wave 2), ss–Social Support (Wave 2), pb–Prosocial Behavior (Wave 2), pr2 –Perceived Risk of COVID-19 (Wave 2), ps3 –Pretraumatic Stress (Wave 3), d3 –Depressive Symptoms (Wave 3), gad3 –Generalized Anxiety Disorder (Wave 3), ss3 –Social Support (Wave 3), pb3 –Prosocial Behavior (Wave 3), pr3 –Perceived Risk of COVID-19 (Wave 3).
(DOCX)
