# Supplementary material for: Predictors of pretraumatic stress during the COVID-19 pandemic in Poland
Source: PLoS One. 2023 Aug 18;18(8):e0290151. doi: 10.1371/journal.pone.0290151 (PMC10437860; doi:10.1371/journal.pone.0290151)
Supplement: S1 Table — *p < = .05 **p < = .01 ps–Pretraumatic Stress, pb–Prosocial Behavior, es–Emotional Stability, ext–Extraversion, agr–Agreeableness, con–Conscientiousness, int–Intellect/Imagination, sd–Social Desirability. (DOCX) [file pone.0290151.s001.docx]

**Table S1. Descriptive statistics (Pilot Study)**

|  | ps | pb | es | ext | agr | con | int | sd |
| --- | --- | --- | --- | --- | --- | --- | --- | --- |
| M | 18.04 | 21.61 | 2.98 | 3.14 | 3.70 | 3.12 | 3.71 | 19.86 |
| SD | 14.55 | 9.48 | 0.78 | 1.04 | 0.74 | 0.87 | 0.63 | 3.21 |
| skewness | 1.115 | -0.05 | 0.05 | -0.17 | -0.32 | -0.19 | -0.19 | -0.30 |
| kurtosis | 0.70 | -0.44 | 0.67 | -0.75 | -0.28 | -0.42 | -0.28 | 0.85 |

***p <= .05 **p <= .01**

**ps – Pretraumatic Stress, pb – Prosocial Behavior, es – Emotional Stability, ext – Extraversion, agr – Agreeableness, con – Conscientiousness, int – Intellect/Imagination, sd – Social Desirability**
