# Supplementary material for: Predictors of pretraumatic stress during the COVID-19 pandemic in Poland
Source: PLoS One. 2023 Aug 18;18(8):e0290151. doi: 10.1371/journal.pone.0290151 (PMC10437860; doi:10.1371/journal.pone.0290151)
Supplement: S2 Table — *p < = .05 **p < = .01 ps–Pretraumatic Stress, pb–Prosocial Behavior, es–Emotional Stability, ext–Extraversion, agr–Agreeableness, con–Conscientiousness, int–Intellect/Imagination. (DOCX) [file pone.0290151.s002.docx]

**Table S2. Correlates of pretraumatic stress (pilot study), controlling for social desirability**

|  | ps | pb | es | ext | agr | con | int |
| --- | --- | --- | --- | --- | --- | --- | --- |
| ps | 1 |  |  |  |  |  |  |
| pb | .23* | 1 |  |  |  |  |  |
| es | -.30** | .08 | 1 |  |  |  |  |
| ext | .05 | .27* | .16 | 1 |  |  |  |
| agr | .24* | .29** | -.17 | .24* | 1 |  |  |
| con | .05 | .14 | .25* | .02 | -.01 | 1 |  |
| int | -.37** | .09 | .25* | .28** | .19* | .08 | 1 |

**p* <= .05 ***p* <= .01

**ps – Pretraumatic Stress, pb – Prosocial Behavior, es – Emotional Stability, ext – Extraversion, agr – Agreeableness, con – Conscientiousness, int – Intellect/Imagination**
