# Supplementary material for: Predictors of pretraumatic stress during the COVID-19 pandemic in Poland
Source: PLoS One. 2023 Aug 18;18(8):e0290151. doi: 10.1371/journal.pone.0290151 (PMC10437860; doi:10.1371/journal.pone.0290151)
Supplement: S3 Table — (DOCX) [file pone.0290151.s003.docx]

**Table S3. Sociodemographic characteristics (main study)**

| Variable | Category | Waves of the study | | | | | |
| --- | --- | --- | --- | --- | --- | --- | --- |
|  |  | First wave  (*N* = 1067) | | Second wave  (N = 894) | | Third wave  (N = 752) | |
|  |  | n | % | n | % | n | % |
| Gender | Female | 542 | 50.8 | 438 | 49.0 | 360 | 47.9 |
|  | Male | 525 | 49.2 | 456 | 51.0 | 392 | 52.1 |
| Education | Primary education | 32 | 3.0 | 25 | 2.8 | 20 | 2.7 |
|  | Vocational training | 95 | 8.9 | 82 | 9.2 | 75 | 10.0 |
|  | Secondary education | 336 | 31.5 | 268 | 30.0 | 224 | 29.8 |
|  | Post-secondary education | 106 | 9.9 | 81 | 9.1 | 67 | 8.9 |
|  | University degree | 498 | 46.7 | 438 | 49.0 | 366 | 48.6 |
