# Supplementary material for: Predictors of pretraumatic stress during the COVID-19 pandemic in Poland
Source: PLoS One. 2023 Aug 18;18(8):e0290151. doi: 10.1371/journal.pone.0290151 (PMC10437860; doi:10.1371/journal.pone.0290151)
Supplement: S4 Table — ps1 –Pretraumatic Stress (Wave 1), d1 –Depressive Symptoms (Wave 1), gad1 –Generalized Anxiety Disorder (Wave 1), ss1 –Social Support (Wave 1), pb1 –Prosocial Behavior (Wave 1), pr 1 –Perceived Risk of COVID-19 (Wave 1), pp 1– Perceived Positive Sides of Pandemic (Wave 1), ps2 –Pretraumatic Stress (Wave 2), d2 –Depressive Symptoms (Wave 2), gad2 –Generalized Anxiety Disorder (Wave 2), ss–Social Support (Wave 2), pb–Prosocial Behavior (Wave 2), pr2 –Perceived Risk of COVID-19 (Wave 2), ps3 –Pretraumatic Stress (Wave 3), d3 –Depressive Symptoms (Wave 3), gad3 –Generalized Anxiety Disorder (Wave 3), ss3 –Social Support (Wave 3), pb3 –Prosocial Behavior (Wave 3), pr3 –Perceived Risk of COVID-19 (Wave 3). (DOCX) [file pone.0290151.s004.docx]

**Table S4. Descriptive statistics (main study)**

|  | ps1 | d1 | gad1 | ss1 | pb1 | pr1 | pp1 | ps2 | d2 | gad2 | ss2 | pb2 | Pr2 | ps3 | d3 | gad3 | ss3 | pb3 | Pr3 |
| --- | --- | --- | --- | --- | --- | --- | --- | --- | --- | --- | --- | --- | --- | --- | --- | --- | --- | --- | --- |
| M | 26.81 | 7.75 | 6.16 | 17.89 | 32.93 | 18.61 | 23.97 | 27.09 | 7.27 | 5.78 | 17.81 | 32.04 | 18.22 | 26.90 | 6.74 | 5.39 | 17.66 | 31.47 | 18.36 |
| SD | 17.71 | 5.68 | 5.15 | 3.75 | 6.58 | 5.20 | 6.45 | 18.05 | 5.98 | 5.28 | 3.76 | 7.17 | 5.53 | 18.34 | 5.71 | 5.16 | 3.80 | 7.26 | 5.58 |
| skewness | 0.21 | 0.59 | 0.66 | -0.11 | -0.22 | -0.23 | -0.18 | 0.15 | 0.68 | 0.82 | -0.18 | -0.33 | -0.20 | 0.10 | 0.65 | 0.79 | -0.11 | -0.25 | -0.25 |
| kurtosis | -0.93 | -0.54 | -0.29 | -0.30 | -0.18 | 0.13 | 0.15 | -1.00 | -0.41 | -0.05 | 0.03 | 0.08 | -0.19 | -1.12 | -0.43 | -0.15 | 0.08 | 0.25 | -0.10 |

**ps1 – Pretraumatic Stress (Wave 1), d1 – Depressive Symptoms (Wave 1), gad1 – Generalized Anxiety Disorder (Wave 1), ss1 – Social Support (Wave 1), pb1 – Prosocial Behavior (Wave 1), pr 1 – Perceived Risk of COVID-19 (Wave 1), pp 1– Perceived Positive Sides of Pandemic (Wave 1), ps2 – Pretraumatic Stress (Wave 2), d2 – Depressive Symptoms (Wave 2), gad2 – Generalized Anxiety Disorder (Wave 2), ss – Social Support (Wave 2), pb – Prosocial Behavior (Wave 2), pr2 – Perceived Risk of COVID-19 (Wave 2), ps3 – Pretraumatic Stress (Wave 3), d3 – Depressive Symptoms (Wave 3), gad3 – Generalized Anxiety Disorder (Wave 3), ss3 – Social Support (Wave 3), pb3 – Prosocial Behavior (Wave 3), pr3 – Perceived Risk of COVID-19 (Wave 3)**
